# Distinguishing programmatic change from data artifacts in hospital certification records: a change‑point and leakage-audit approach in Thailand

**DOI:** 10.1080/16549716.2026.2758984

**Published:** 2026-11-11

**Authors:** Sakul Sinchai, Ananta Sinchai

**Affiliations:** a Faculty of Public Health, Bangkok Thonburi University, Bangkok, Thailand; b School of Integrated Innovative Technology, King Mongkut’s Institute of Technology Ladkrabang, Bangkok, Thailand

**Keywords:** Routine health information systems, data quality, feature leakage, change‑point detection, clustering analysis

## Abstract

**Background:**

Routine Health Information Systems (RHIS) produce administrative data where apparent shifts may reflect recording practices rather than genuine program changes. Methods appearing to support such shifts can share hidden information with them, a reproducibility concern in machine‑learning research.

**Objective:**

This study aims to illustrate a leakage pattern that can produce misleading corroboration in change‑point and clustering analyses of routine health data and to propose a protocol to reduce such circularity. This is a preliminary, proof-of-concept validation, not a full operating-characteristic evaluation.

**Methods:**

Certification records from 86 hospitals in a regional environmental‑certification program in Thailand were analysed. Change‑point detection on a recorded outcome field was assessed against three checks: an alternative construct, an alternative definition, and clustering restricted to timing-unrelated features. The protocol was also tested on synthetic data with a known, non‑leaking discontinuity.

**Results:**

A discontinuity was detected in the recorded outcome field, consistent across two change‑point algorithms. It was not supported by the alternative construct or definition and was not reproduced by leakage‑free clustering. An initial clustering analysis appeared to corroborate it but later shared timing information. All three checks were consistent with the known discontinuity in the synthetic dataset.

**Conclusion:**

The discontinuity is more consistent with a change in recording or definitional practice than with a genuine program effect, though the data cannot confirm the mechanism. This preliminary protocol may give analysts a practical, low‑cost safeguard against reporting such shifts as genuine change without further checks. It could be automated as a first-pass screen pending further validation.

## Paper Context

- **Main findings:** A sharp apparent rise in recorded certification upgrades in a Thai regional hospital certification program did not hold up under independent checks, suggesting it reflects how outcomes were recorded rather than genuine improvement in hospital performance.
- **Added knowledge:** This study identifies a case where an analysis method can appear to confirm a data pattern by relying on information already tied to it, not independent evidence, and provides preliminary evidence, using data with a known genuine change, that a corrected version of the method still correctly detects real change when one is present.
- **Global health impact for policy and action:** Programs relying on routine administrative records to track performance, in Thailand and in similar settings, could use this low-cost check to avoid mistaking a recording change for a genuine one before acting on it.

## Background

The Green and Clean Hospital (GCH) program is a national, voluntary environmental-management policy administered by Thailand’s Ministry of Public Health (MOPH), certifying hospitals at tiers from Standard to Excellence [1]. Regional health offices record a binary renewal-outcome field, upgraded or maintained, alongside certification tier, generating continuous, routine administrative data. Health Region 11 comprises seven southern provinces: Nakhon Si Thammarat, Krabi, Phang-nga, Phuket, Surat Thani, Ranong, and Chumphon. This administrative structure is broadly similar to that of many national health programs and is the setting for the case study in this paper.

Machine learning and change-point detection are increasingly applied to administrative and program-monitoring data, outside the surveillance contexts where these methods originated. In brief, change-point detection flags where the pattern of a time-ordered sequence shifts, and clustering groups similar records together without using outcome labels. This creates a specific risk that has received little attention in this literature. A corroborating analysis may appear independent when its clustering supports a change-point finding yet shares an input feature proxying the variable driving the result. This is a specific instance of data leakage: the introduction of target information not legitimately available to the analysis [2]. Leakage has been identified as a cross-disciplinary driver of reproducibility failures in machine-learning-based science, affecting at least 329 papers across 17 research fields [3]. In health care, label leakage, contamination of model inputs with outcome information, is a recognised pitfall in clinical prediction modelling [4]. Most documented cases involve supervised prediction. Whether the same failure compromises change-point-plus-clustering pipelines, a combination that appears increasingly common in applied health-data-science work, has not been demonstrated with a real, quantified case.

Separately, RHIS generate continuous administrative data for program monitoring. Apparent shifts in a recorded indicator can reflect a change in recording practice rather than in the measured phenomenon [5,6]. Standard RHIS quality assurance relies on periodic manual review against expected patterns [7]. This approach has identified data-quality problems in national health-management systems, including inconsistencies invisible to routine reporting [8]. Interrupted time-series methods have evaluated data-quality interventions on Health Management Information System (HMIS) accuracy [9]. Whether lightweight, non-circular checks can complement manual RHIS review, rather than evaluate interventions, remains untested.

Two further literatures bear on this diagnostic problem. First, change-point methods for categorical and ordinal time series must account for temporal dependence. Li and Lu (2022) [10] showed that ignoring this dependence can bias change-point estimates, a risk for any binary outcome ordered by certification date. Exact binomial tests, such as Ellenberger et al. (2021) [11], add power in small samples. Neither approach asks whether a detected shift reflects the underlying process or an artifact of how the sequence is timed, the central question of this study. Second, a result derived from the data that generated a hypothesis cannot independently test it. Kriegeskorte et al. (2009) [12] termed this circular analysis, or double dipping, in the context of neuroimaging. We adopt their standard for independence directly. An analysis counts as independent corroboration only when its inputs share no deterministic or near-deterministic link to the certification date. Clustering on date-derived features, such as program time or certification quarter, repeats the same test under a different name. The Adjusted Rand Index (Hubert and Arabie, 1985) [13], applied in *Clustering*, measures clustering validity but cannot establish this independence.

This study addresses both gaps in a single case: change-point detection and clustering applied to Green and Clean Hospital certification records from Health Region 11 in Thailand. A naive clustering corroboration of a change-point result proves to be leakage, in the Kaufman et al. (2012) [2] sense, arising from a shared date-derived feature. We present a corrected, non-circular protocol with a preliminary, proof-of-concept validation, detailed in the Materials and methods.

## Objectives

1. This study aims to demonstrate an undocumented feature-leakage instance in a change-point-plus-clustering pipeline, using a real routine health dataset. The leakage occurs when an apparently independent corroborating analysis shares a date-derived input with the change-point target.
2. This study also aims to develop a non-circular diagnostic protocol that detects this failure mode and distinguishes a genuine discontinuity from a recording artifact. It combines an alternative-construct check, a leakage-free clustering re-analysis, and a preliminary, proof-of-concept validation.
3. This study further aims to support the protocol using a synthetic dataset with a known, genuine, non-leaking discontinuity. This tests whether it correctly corroborates a real effect rather than uniformly failing to corroborate any input.

## Methods

### Study design

This is a retrospective, descriptive, analytical study of secondary, aggregate administrative data that develops a diagnostic protocol with preliminary validation against a real and a synthetic case. No patient-level or individually identifiable information was used, and no primary data were collected from hospitals or individuals. No internal program records or personal communications beyond the administrative dataset in this study were used.

### Study setting

The study setting is Health Region 11, one of thirteen health regions administered by Thailand’s Ministry of Public Health, comprising the same seven southern provinces named earlier. Regional health offices there coordinate provincial-level implementation of centrally issued MOPH programs, including the GCH certification scheme [14].

### Data source

The dataset comprised administrative records for 86 hospitals in the GCH certification program across the seven provinces of Health Region 11, extracted as of 31 March 2026. Available fields were a hospital identifier (hoscode), province code, certification tier (Standard, Challenge, Challenge 2, or Excellence), certification date, certificate expiry date, and a renewal-outcome field. This field recorded whether the hospital was upgraded or maintained relative to its prior tier. Certification tier follows the hierarchy codified by the Department of Health [14]. No sampling was performed. The dataset represents all hospitals with an active or recently expired GCH certification record in the region. Each of the 86 hospitals contributes exactly one certification record to this dataset, its most recent renewal as of the extraction date. No hospital appears more than once, so the dataset contains no repeated measurements and no panel structure. Supplementary Table S1 lists each hospital’s anonymized identifier, certification date, and recorded outcome, confirming this one-record-per-hospital structure.

### Data preparation

Certification and expiry dates were parsed to compute certificate duration in days. One record showed an implausible negative duration (−1 day), retained but flagged as a likely data-entry error. As a sensitivity check, we repeated the leakage-free clustering (k = 2 to 5) excluding this record. The Adjusted Rand Index against the change-point grouping changed by at most 0.04 across all four solutions and stayed in the same weak-or-no-agreement range either way. Excluding the record removes essentially all of the variance in the standardized duration variable. Its standard deviation falls from 78.9 to 3.4 days once the record is removed. The remaining 85 hospitals’ certificate durations then cluster tightly between 729 and 760 days; the Limitations section returns to this property. Certification tier was ordinally encoded (Standard = 1, Challenge = 2, Challenge 2 = 2.5, Excellence = 3). The renewal-outcome field is recorded as a single categorical value per hospital at each renewal. Its precise operational definition could not be independently verified, nor could we confirm consistent application across the study period. This uncertainty motivates the protocol herein and is central to our results.

### Feature construction

We derived several features from available administrative fields, with no external data linkage: certification month, quarter, year, certificate duration, province code, and certification tier. We also derived program time: days elapsed since the earliest certification date. Program time, certification quarter, and certification month are all deterministic functions of certification date, the source of the leakage instance diagnosed and corrected. Figure 1 summarizes this pipeline. Certification date orders the change-point analysis and generates the derived features used in the corroboration analyses. A feature correlated with certification date is therefore a threat to independent corroboration, addressed directly later in *Clustering*. Figure 2 summarizes the analytical framework: an initial change-point result is never sufficient by itself but carried forward as a provisional candidate and tested against a four-part framework. The four parts are algorithmic robustness, an alternative outcome definition, an alternative or related construct, and a leakage-free secondary analysis. Each is described in detail hereafter. Only a candidate breakpoint that survives all four checks is interpreted as evidence of a genuine discontinuity; one that does not is treated as a likely recording or definitional artifact.

**Figure 1.**
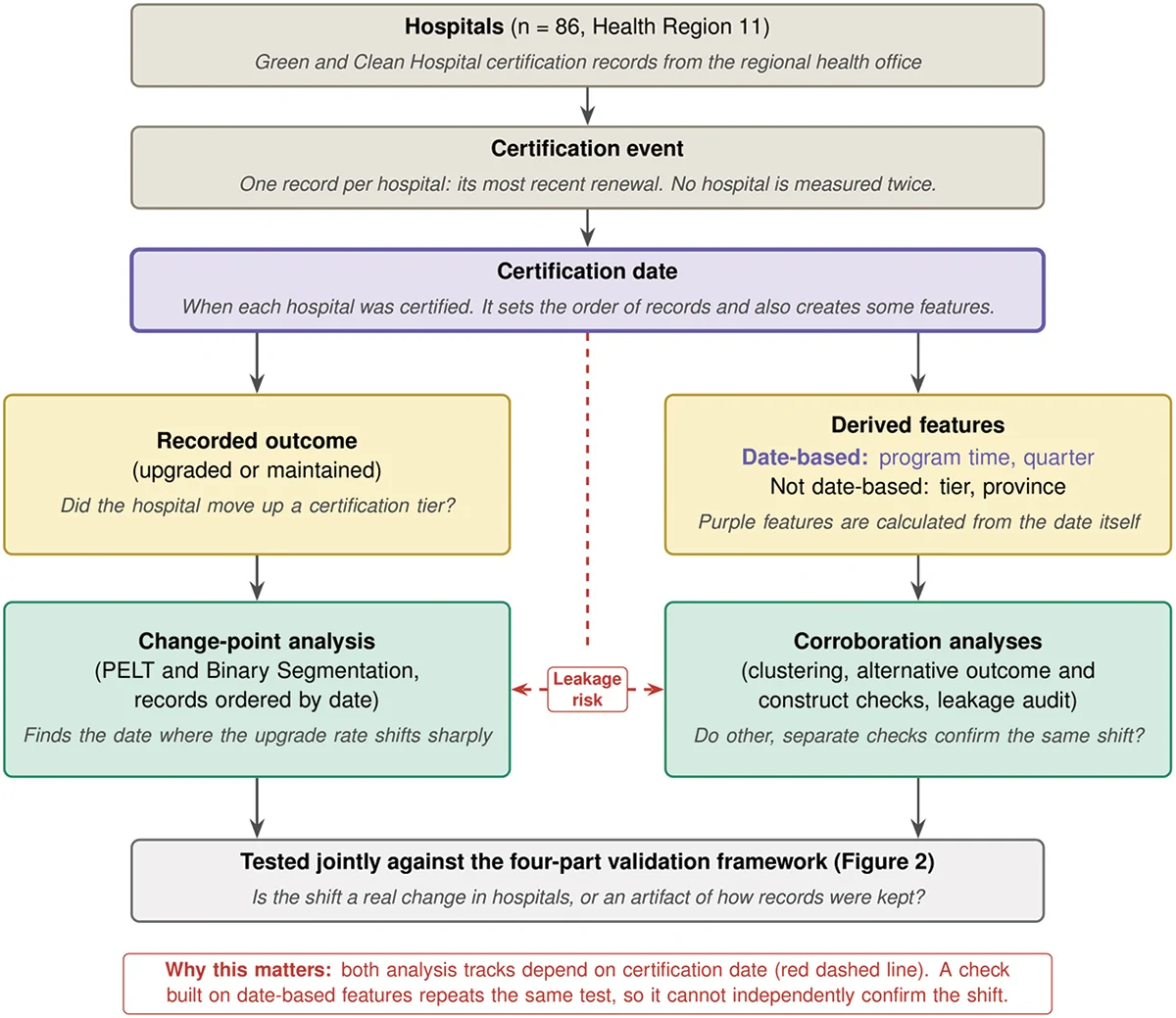
Data pipeline from a raw administrative record to the two analysis tracks used in this study.

**Figure 2.**
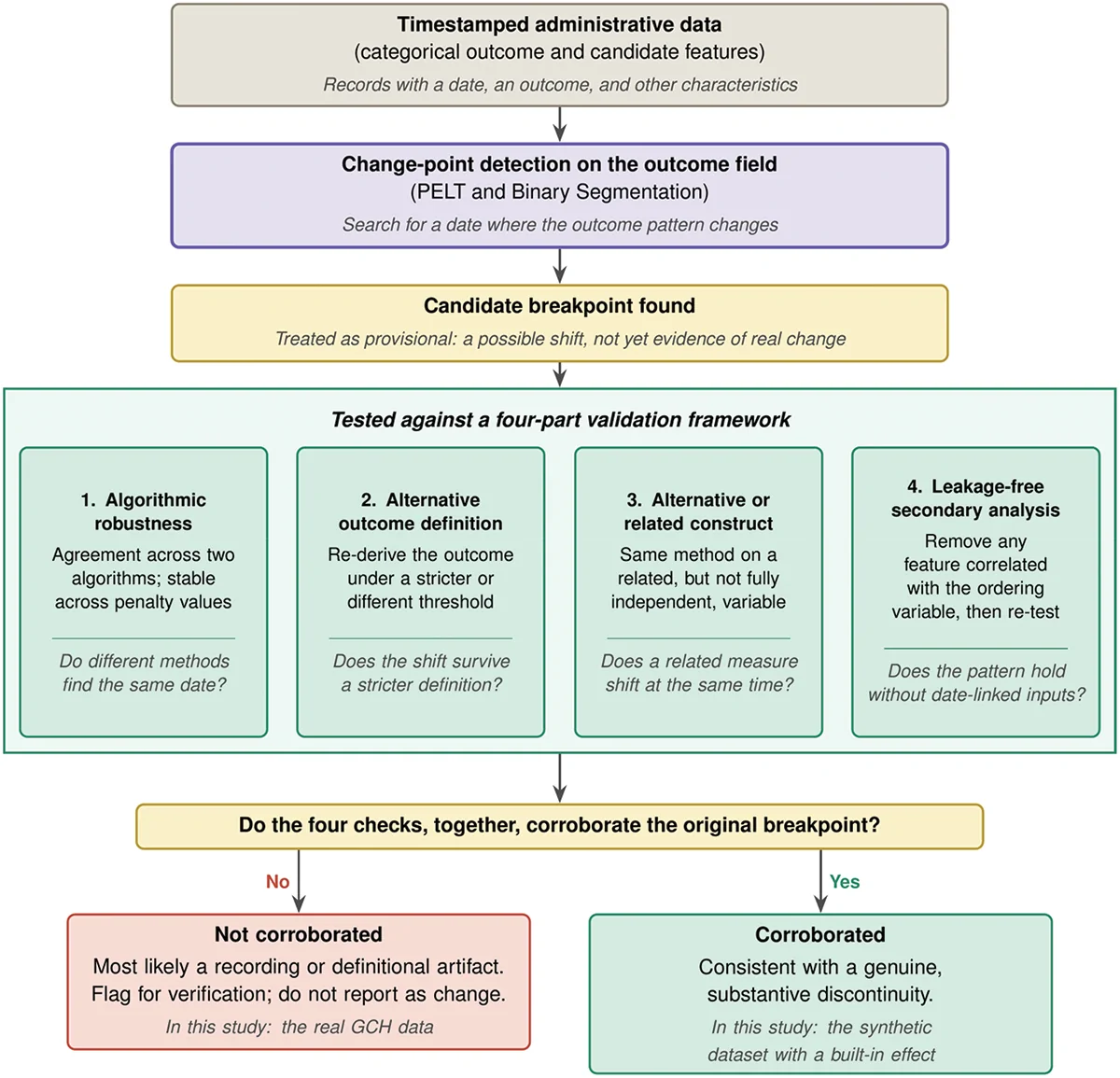
Analytical framework: an initial change-point finding is treated as provisional and tested against a four-part validation framework before interpretation.

### Change-point detection

We treated renewal outcomes (upgraded = 1, maintained = 0) as a one-dimensional signal, ordered by certification date. We applied the Pruned Exact Linear Time (PELT) algorithm [15,16] with a radial basis function (RBF), implemented in the ruptures Python library [17]. As a robustness check, we also applied Binary Segmentation with an L2 cost [17], constrained to a single breakpoint. We also tested how sensitive PELT was to the penalty parameter across seven values (1, 2, 3, 5, 8, 10, and 15). We then compared upgrade proportions before and after the identified breakpoint using Fisher’s exact test [18]. We used an RBF cost function because it detects a broad class of distributional shifts in the mean of a bounded 0/1 signal without assuming a specific parametric form. A cost function derived from a specific distribution would impose that assumption instead. We considered, but did not adopt, an explicit Bernoulli or binomial change-point model, which can offer higher statistical power in small samples with a binary outcome [11]. We used the more general RBF approach because our aim was a distribution-agnostic first-pass screen applicable across different RHIS indicators, not a model tailored to one outcome type. A binomial-specific method would be more tailored to this binary outcome, so we ran a Bernoulli likelihood-ratio scan as a supporting check. Because certification events are irregularly spaced in calendar time, we ordered the signal by event sequence rather than calendar date. The detected breakpoint therefore marks the 31st event in sequence, not a fixed calendar date. As a check on this choice, we also aggregated the outcome data by calendar month and reran PELT on the resulting monthly upgrade-rate series. This calendar-time analysis identified a change point in January 2025, roughly three months after the event-order breakpoint of 20 September 2024. This discrepancy shows the detected breakpoint depends on how time is represented, by event order or by calendar time. We treat this sensitivity as a further reason to treat the initial discontinuity as provisional, pending the further robustness checks reported later in the subsequent section.

### Robustness checks for outcome-variable stability

Agreement between two change-point algorithms suggests that a breakpoint is not an artifact of a single method. Stability across penalty values provides similar evidence for the tuning choice. Neither establishes that the breakpoint reflects genuine performance change rather than a recording change. This is the first protocol step: treat algorithm agreement as necessary but not sufficient. We organize the remaining checks into three further categories, alongside this algorithmic-robustness step, forming the four-part validation framework. These are an alternative outcome definition, an alternative or related construct, and a leakage-free secondary analysis, described in *Clustering*. We use ‘alternative or related construct,’ not ‘independent construct,’ because certification tier and the recorded renewal outcome both derive from the same underlying certification decision. They are not statistically independent measurements, though they are recorded through separate program fields and could in principle diverge. First, we applied the same PELT procedure to the ordinal certification-tier series rather than to the binary outcome field. Tier is a more direct, objective certification-status measure than a locally recorded upgrade-or-maintain label. Second, we constructed a stricter alternative outcome, coding a hospital as upgraded only if it held Challenge 2 or Excellence tier, and comparing this proportion across the breakpoint. If the discontinuity is a stable certification-status property, both checks should show a comparable shift at a comparable date. If instead it reflects recording, they may show no shift, or a shift at a different time. The original breakpoint remains statistically robust on its own terms.

### Clustering

Available features mixed categorical (province and certification quarter) and numeric (certification tier, program time, certificate duration) variables. We initially used k-prototypes clustering [19,20], extending k-means to mixed-type data [21], on the full feature set with certification tier excluded. This recovered a split visually resembling the change-point result, initially read as independent corroboration. Formally, the ARI [13] between this clustering and the change-point grouping was only 0.147 at k = 2, a weak level of agreement by conventional benchmarks. This weak numerical agreement, well below what the visual impression suggested, is the leakage instance central to this study. Program time is a deterministic function of certification date; the renewal-outcome field splits almost perfectly by date at the breakpoint. Any clustering method given program time or certification quarter as an input is therefore likely to recover a similar split. This may occur regardless of unrelated differences. Per Kaufman et al. (2012) [2], program time is a leaking feature, carrying certification-date-linked target information that should not have been treated as a validating input. We use ‘feature leakage’ broadly, following Kaufman et al. (2012), where leakage denotes information that should not be legitimately available to a validating analysis. Although our clustering is unsupervised and does not use the renewal outcome as an input, its features, program time and certification quarter, are deterministic functions of certification date. The change-point analysis likewise orders the outcome by certification date. Thus, both analyses share their key organizing variable, creating leakage through the shared ordering variable rather than the outcome label. We report both analyses for transparency. The second step of our protocol repeats the clustering using only features unrelated to certification date: province and certificate duration. Numeric features were standardized; cluster number was selected using the elbow method (k = 2 to 6), Cao initialization, and multiple random restarts (n_init = 15). We report k = 2 as the most interpretable, with k = 3 to 5 as a robustness check. This corrected analysis is the only result treated as a genuine, non-circular test of grouping independent of certification timing.

### Discarded predictive-classification approach

Before finalizing the protocol, we evaluated a supervised-classification alternative: predicting the renewal-outcome field from the same features with Random Forest and logistic-regression classifiers [22], validated with leave-one-out cross-validation (LOOCV). Both classifiers achieved apparently strong performance: accuracy of 92 to 93%, and area under the receiver operating characteristic curve (AUC) of 0.94 to 0.96. A single univariate decision rule, based solely on certification date, achieved comparable or higher accuracy (94.2%) than either multivariate classifier.

This is a second, independent instance: certification date, or a close proxy, was available to a model whose target was almost perfectly determined by certification date. The classifiers reflected this shared information, not a generalizable relationship between hospital characteristics and outcome. A threshold rule reducing to ‘certified after a given date’ offers no actionable prediction for program managers. We report this discarded analysis alongside the clustering correction because both reveal the same leakage pattern, visible only upon re-examination of each analysis against its outcome. This is a systematic risk in this pipeline class, not an isolated oversight.

### Synthetic validation of protocol sensitivity

The GCH case illustrates that our protocol correctly identified a discontinuity as unsupported by independent checks, a true negative, in this instance. It does not show that the protocol can identify a genuine discontinuity as supported, a true positive. Otherwise, a protocol that never corroborates anything would trivially agree with the GCH result, making that finding uninformative. We therefore constructed a synthetic dataset of 86 hospitals, matching the real sample size, province distribution, and certification-date range, with a genuine, built-in discontinuity. Certification dates were drawn independently of hospital attributes. Each was assigned a latent class, unrelated to certification date, determining typical certificate duration and a modest, persistent upgrade probability effect. A genuine change in upgrade probability was then introduced at a specific date, representing a policy event independent of the latent class. We ran the identical protocol on this synthetic dataset. It included change-point detection on the binary outcome, the alternative-construct check with a correlated ordinal tier variable, the stricter alternative-definition check, and leakage-free clustering using province and duration [23]. Figure 3 shows the synthetic timeline, with the built-in discontinuity visible in the simulated tier values near the breakpoint; Figure 4, the real GCH data, shows no comparable shift.

**Figure 3.**
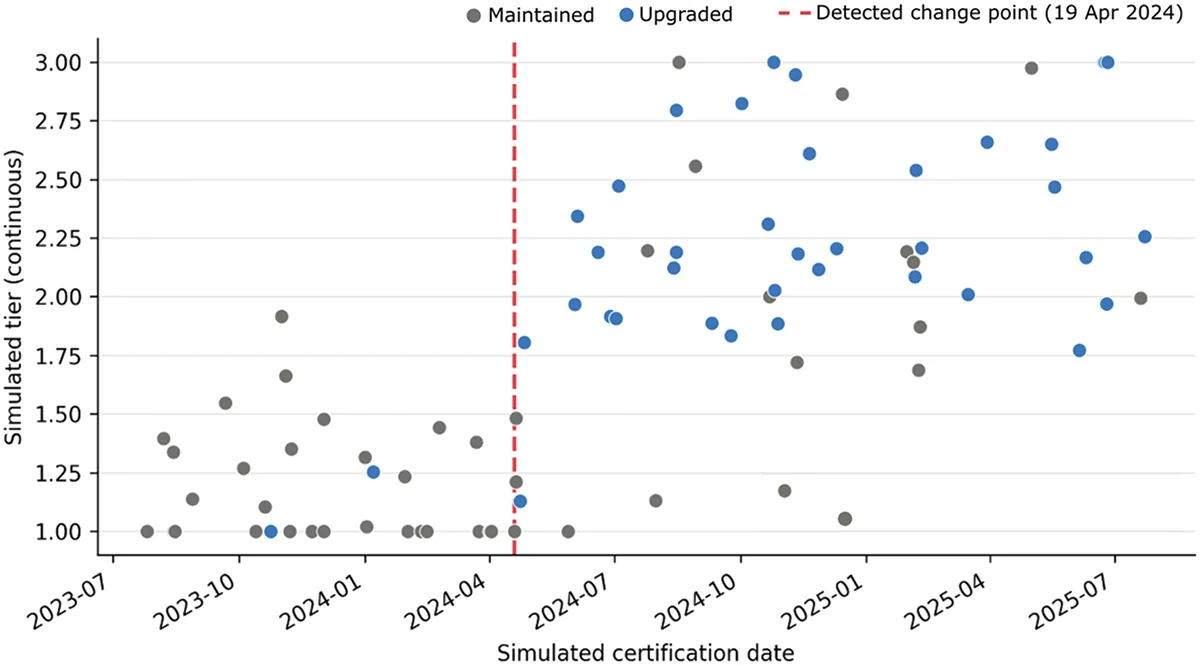
Synthetic dataset with a genuine, built-in discontinuity, used to test whether the protocol can also corroborate a real effect.

**Figure 4.**
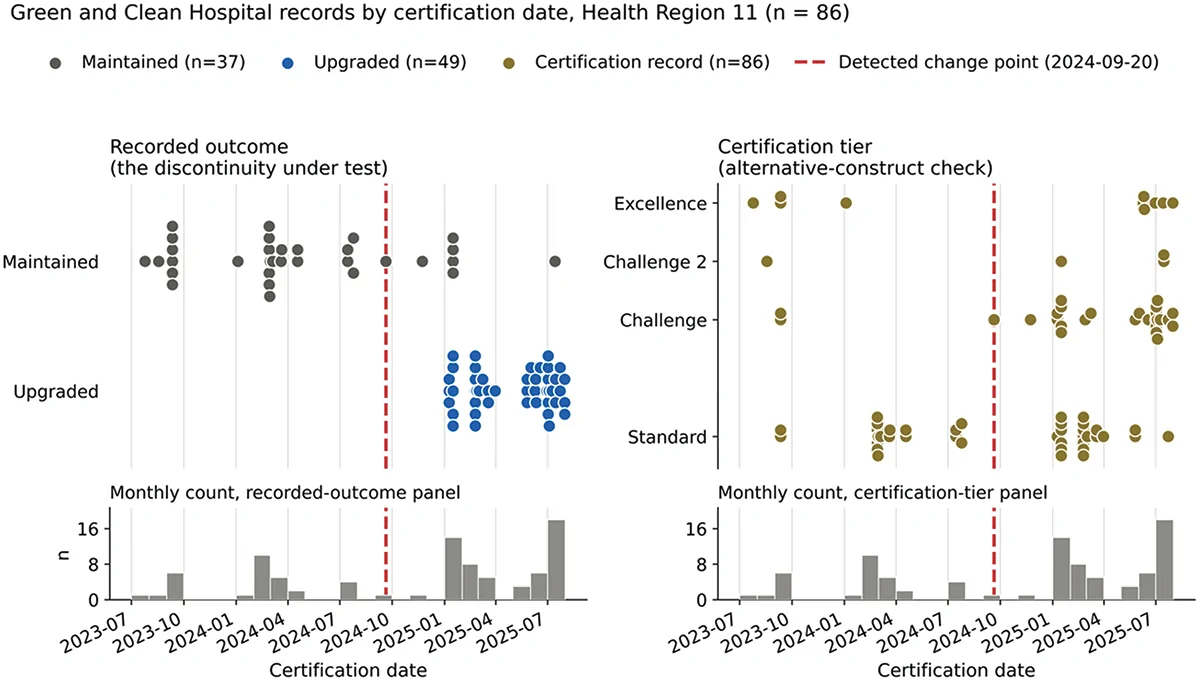
Green and Clean Hospital records by certification date, Health Region 11 (*n* = 86). Left: recorded outcome (maintained or upgraded). Right: certification tier (standard to Excellence), on its own axis. Each panel is paired below with its own monthly count of certification records, aligned to the same axis. Points close in time are offset vertically for visibility; the offset carries no other meaning.

### Preliminary multi-scenario synthetic check

The prior single synthetic example illustrates the protocol’s behaviour on one data-generating process. To assess this behaviour more systematically, we generated data under six scenarios, run for 50 replicates each with fixed random seeds for reproducibility. Together, these scenarios test whether the protocol stays silent with no effect, corroborates a genuine effect, and withholds corroboration for a pure artifact. The remaining scenarios test whether it stays stable under multiple discontinuities, a smaller sample, or a gradual rather than a sharp shift. The six scenarios were no discontinuity, a genuine non-date-linked discontinuity, a pure recording artifact tied only to date, and a gradual trend rather than a sharp break. The remaining two scenarios added two genuine discontinuities in the same series, and the genuine-discontinuity case repeated at a smaller, more temporally imbalanced sample of 40 hospitals.

In every scenario, certification dates were drawn independently of any hospital attribute, and province was drawn to match the real data’s distribution. Each simulated hospital was assigned a latent class, unrelated to date, drawn from a Bernoulli distribution with probability 0.35. This class determined certificate duration, drawn from a normal distribution with a class-specific mean, and contributed a small, persistent effect on upgrade probability. Certification tier and upgrade probability were then built from a scenario-specific base signal, a step, a ramp, or a constant, plus this class effect and independent random noise.

For each replicate, we ran the same protocol used on the real data. We recorded whether it corroborated any detected breakpoint, using a predefined rule: a comparable tier shift together with a comparable leakage-free clustering spread. Both thresholds were set from a separate pilot run, not the replicates used for the reported results, and are specific to this data-generating process and sample size. We report this as an explicitly preliminary, illustrative check, not a full operating-characteristic study.

### Software

All analyses used Python 3.12, with pandas and scikit-learn for data handling and cross-validation [22,24], ruptures [17] for change-point detection and kmodes for k-prototypes clustering [19,20]. Matplotlib supported visualization and scipy supported statistical testing.

## Results

### Sample characteristics

The dataset comprised 86 hospitals across the seven provinces of Health Region 11, with 4 to 24 hospitals per province (Table 1). Certification tier distribution was: Standard, 44 hospitals (51.2%); Challenge, 28 (32.6%); Challenge 2, 4 (4.7%); and Excellence, 10 (11.6%). Overall, 49 hospitals (57.0%) were recorded as upgraded at their most recent renewal, and the remaining 37 (43.0%) as maintained. Certification dates spanned 25 July 2023 to 31 July 2025.

**Table 1.** Sample characteristics of 86 hospitals with Green and Clean Hospital certification, Health Region 11.

Characteristic	n	%
Total hospitals	86	100.0
Certification tier: Standard	44	51.2
Certification tier: Challenge	28	32.6
Certification tier: Challenge 2	4	4.7
Certification tier: Excellence	10	11.6
Recorded outcome: Upgraded	49	57.0
Recorded outcome: Maintained	37	43.0
Province: Nakhon Si Thammarat (80)	24	27.9
Province: Krabi (81)	9	10.5
Province: Phang-nga (82)	9	10.5
Province: Phuket (83)	4	4.7
Province: Surat Thani (84)	24	27.9
Province: Ranong (85)	5	5.8
Province: Chumphon (86)	11	12.8

### Change-point detection

Both PELT and Binary Segmentation identified an identical breakpoint [15,17], falling at the 31st certification event, corresponding to 20 September 2024 (Figure 4). This was stable across all seven tested PELT penalty values; only the lowest penalty (pen = 1) flagged a second, weaker candidate breakpoint, unsupported by Binary Segmentation. A Bernoulli likelihood-ratio scan supported this, placing the break two records later within the same sparse interval (0 of 32 versus 49 of 54; permutation *p* < 0.001). The recorded upgrade rate differed sharply across the breakpoint: 0% (0 of 30 hospitals) before 20 September 2024, versus 87.5% (49 of 56; Table 2) after. Fisher’s exact test found this unlikely to occur by chance (Fisher (1922) [18]; odds ratio not estimable due to a zero cell; *p* = 8.0 × 10^−17^). Because one cell is zero, this *p*-value is uninformative about the size of the difference, so we report exact Clopper-Pearson confidence intervals for each proportion instead. These are 0.0% (95% CI 0.0–11.6%) before the breakpoint and 87.5% (95% CI 75.9–94.8%) after it. The Newcombe hybrid-score interval for the difference between these two proportions spans +81.2 to +100.0% points, excluding zero throughout. Because each hospital contributes a single record, this comparison of hospitals before and after the breakpoint is necessarily a comparison of two different groups, not the same hospitals measured twice. By the algorithm-agreement criterion, this is an encouraging result. As set out in *Robustness checks for outcome-variable stability*, that criterion is only the first protocol step, not the conclusion. Figure 4 presents the recorded outcome and certification tier in separate panels. The left panel shows the recorded outcome, the discontinuity under test. The right panel shows certification tier, the alternative-construct check, on its own axis. Before the change point, recorded outcomes are almost entirely maintained. After it, they are almost entirely upgraded. Certification tier shows no comparable break at the same date. Each panel is paired with its own monthly count of certification records below it, so observation density around the change point is visible directly beneath each panel.

**Table 2.** Recorded outcomes and tier composition before and after the detected change point (20 September 2024).

Period	n hospitals	Upgraded, n (%)	Standard tier (%)	Challenge tier (%)
Before 20 September 2024	30	0 (0.0)	76.7	6.7
On/after 20 September 2024	56	49 (87.5)	37.5	46.4
Fisher’s exact test	n/a	p = 8.0 × 10^−17^	n/a	n/a

### Robustness of the outcome-variable discontinuity

Table 3 summarizes the alternative-construct checks. Applying PELT to the ordinal certification-tier series identified breakpoints at 28 February 2024 and 26 May 2025, not 20 September 2024. Mean tier rose only modestly, from 1.383 to 1.759 (1-to-3 scale). Under the stricter alternative definition (upgraded only if Challenge 2 or Excellence tier), the proportion was 16.7% before 20 September 2024 and 16.1% after indicating no shift. Neither alternative-construct check corroborates the original breakpoint.

**Table 3.** Robustness checks on the recorded discontinuity: certification tier and an alternative outcome definition.

Check	Before 20 September 2024	After 20 September 2024	Same breakpoint?
Recorded outcome field (original)	0.0%	87.5%	Yes (by construction)
Change-point on tier series directly	n/a	n/a	No (28 February 2024; 26 May 2025)
Mean certification tier (1–3 scale)	1.383	1.759	No independent break at this date
Stricter definition (tier ≥ Challenge 2)	16.7%	16.1%	No shift

### Clustering results

When certification tier was included as a clustering input alongside program time [20], separating hospitals largely along program time and recovering a split resembling the change-point result. This agreement is leakage, detailed in *Clustering*. Table 4 reports the corrected, leakage-free clustering, using only province and certificate duration. We use the ARI [13] as a predefined, formal similarity criterion throughout, following conventional benchmarks. ARI values of 0.5 or above indicate strong agreement, 0.2 to 0.5 moderate, 0.05 to 0.2 weak, and below 0.05 no meaningful agreement. By this criterion, the corrected clustering showed no meaningful agreement with the change-point grouping at k = 2 (ARI = −0.013). Solutions at k = 3 to 5 showed only weak agreement (ARI = 0.150, 0.100, and 0.098, respectively). The k = 2 solution produced two clusters: 62 hospitals with a 53.2% upgrade rate, and 24 with a 66.7% rate. The first spanned 25 July 2023 to 31 July 2025, the second 18 August 2023 to 23 July 2025. Both clusters span nearly the entire study period, with upgrade rates differing only modestly. Solutions at k = 3 to 5 similarly failed to recover a cluster near 0% or 87.5%, or confined to a narrow date range. Figure 5 plots the two corrected clusters, with upgrade rates close together, versus the earlier 0% to 87.5% split, visually consistent with no comparable split emerges once leaking features are removed.

**Figure 5.**
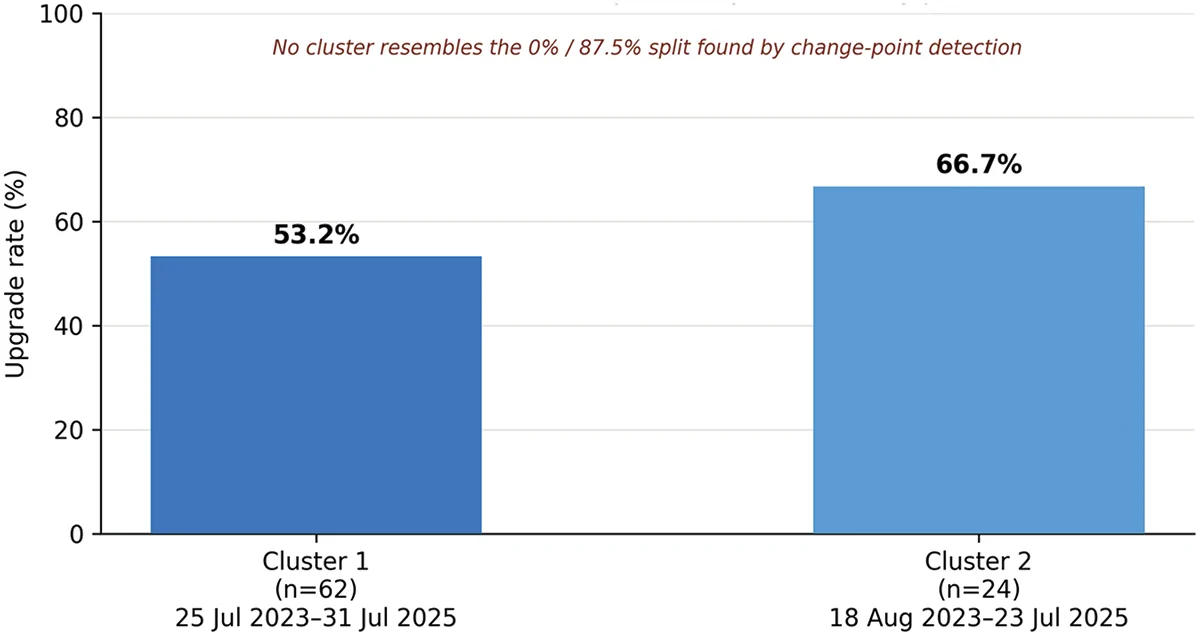
Corrected clustering results: upgrade rate by cluster using non-temporal features only. Unlike the uncorrected analysis, no split resembling the original breakpoint emerges.

**Table 4.** Corrected clustering using only province and certificate duration (features independent of certification date).

Cluster	n	Date range	Upgrade rate (%)
Cluster 1	62	25 July 2023 – 31 July 2025	53.2
Cluster 2	24	18 August 2023 – 23 July 2025	66.7

### Provincial distribution

Certifications were not evenly distributed across provinces before and after the original breakpoint (Table 1). Surat Thani (provincecode 84) and Nakhon Si Thammarat (provincecode 80) contributed disproportionately: 20 and 16 hospitals, respectively, out of 56, or 64.3% of certifications after 20 September 2024, versus 40.0% before. Phang-nga showed the opposite pattern: a larger pre-breakpoint certifications (6 of 30, 20.0%) than post-breakpoint certifications (3 of 56, 5.4%). We treat this provincial pattern as descriptive, possibly reflecting uneven reporting practices rather than a genuine, staggered rollout.

### Synthetic validation: does the protocol also corroborate a genuine effect?

Table 5 summarizes the synthetic validation. Both PELT and Binary Segmentation identified an identical breakpoint at 19 April 2024, close to the true intervention date. The upgrade rate rose from 6.7% before this date to 66.1% after it (*p* = 5.3 × 10^−8^; Figure 3). Unlike the real GCH case, both alternative-construct checks now corroborate this breakpoint. The tier-series change-point fell at 28 May 2024, within five weeks of the outcome breakpoint, with mean tier rising from 1.195 to 2.138. The stricter alternative definition also showed a genuine shift, 0% to 26.8%. The leakage-free clustering, using only province and certificate duration, recovered a clear two-cluster split: one held 31 hospitals with a 58.1% upgrade rate, the other with 38.2%. The first corresponds to 97% of the higher-probability latent class, the second almost entirely to the remaining class. This split does not follow a simple date pattern. Both clusters span nearly the full study period. The clustering result reflects the latent class structure, not certification timing; by construction, no defining feature of either cluster relates to certification date. All three checks that failed to corroborate the real GCH discontinuity corroborate this synthetic, built-in effect.

**Table 5.** Protocol validation summary: real GCH data (artifact case) versus synthetic data (genuine-effect case).

Check	Real GCH data (artifact case)	Synthetic data (genuine-effect case)
Independent-construct (tier) change-point	Different dates; no match	Matches within 5 weeks
Mean tier shift across breakpoint	Modest (1.38 → 1.76)	Substantial (1.20 → 2.14)
Stricter alternative definition	No shift (16.7% → 16.1%)	Real shift (0% → 26.8%)
Leakage-free clustering	No split recovered	Clear split recovered, matching true latent groups

## Discussion

### Preliminary multi-scenario synthetic validation

Across 50 replicates per scenario, the protocol behaved as expected in five of six scenarios. In the ‘none’ scenario, with no built-in discontinuity, PELT found no breakpoint in any replicate, so no corroboration decision arose. In the ‘genuine’ scenario, PELT found a breakpoint in every replicate, and the protocol corroborated it in 84% of these. In the ‘artifact’ scenario, a discontinuity present only in the recorded label, PELT again found a breakpoint in every replicate. The protocol correctly withheld corroboration in all of them, a 0% rate. In the ‘multi_cp’ scenario, with two genuine discontinuities, PELT found a breakpoint in every replicate. The protocol corroborated it in 92% of these, similar to the single-discontinuity case. In the smaller, more imbalanced ‘small_n’ scenario, with 40 hospitals, PELT found a breakpoint in 52% of replicates, lower than at *n* = 86. It corroborated the breakpoint in 50% of the replicates where one was found. Scenario-level results are provided as Supplementary Table S2.

The ‘gradual’ scenario is the one exception worth discussing directly. PELT found a breakpoint in 80% of these smooth-trend replicates, and the protocol corroborated it in 85% of those found. This is a similarly high corroboration rate to the genuine sharp-break scenario, and we do not consider this a failure of the protocol. A gradual trend in this data-generating process still carries a real, non-date-linked tier and clustering signal. The protocol is designed to distinguish a real effect from a pure recording artifact, not a sharp break from a gradual one. It therefore correctly treats a genuine gradual change as corroborated, just as it treats a genuine sharp change.

### Methodological implications

The central methodological finding is that a clustering analysis can appear to independently corroborate a change-point result while sharing the exact information that drove the original result. This is not a hypothetical risk. Our initial analysis committed this error, caught through a deliberate audit of what each method’s inputs shared with the target. Kapoor and Narayanan’s (2023) [3] survey describes this broader pattern. To our knowledge, this study presents a specific, previously undocumented instance of that pattern. It arises in a pipeline that pairs change-point detection with clustering, a combination not covered in their survey.

The substantive GCH finding is more cautious than an uncorrected analysis suggests. The renewal-outcome field shows a sharp, algorithm-stable discontinuity around 20 September 2024. This part of the finding appears robust to algorithm and penalty choice. It is not evidence that hospital performance in Health Region 11 changed abruptly. Two further checks (certification tier, a stricter ‘upgraded’ definition) and the corrected, leakage-free clustering all failed to recover a comparable shift or grouping. We therefore do not interpret it as confirmed evidence of a real, cohort-wide performance change. The interpretation is most consistent with a change in how the outcome field was recorded or defined around September 2024. Without internal Health Region 11 program records, correspondence, or personnel, the data cannot confirm the mechanism or a specific triggering event. We recommend that regional program administrators verify this directly against internal records.

A finding of non-corroboration is informative only if the protocol can also corroborate a genuine effect. The synthetic validation shows that it can, so the protocol distinguished a real effect from an artifact in this preliminary test.

For RHIS practice, this study suggests that the protocol may be a viable, low-cost complement to periodic manual data-quality review [5,7]. The protocol combines algorithm agreement, an alternative-construct check, and a leakage-audited secondary method, unlike periodic review such as Ethiopia’s national HMIS [8]. This gap matters at scale: District Health Information Software 2 (DHIS2) is a widely deployed RHIS used across dozens of low- and middle-income countries, including Kenya [25]. These platforms generate timestamped, categorical records that this protocol can analyse, and interrupted time-series methods have evaluated data-quality interventions [9]. This preliminary protocol targets an earlier workflow point: an automatable first-pass screen for any timestamped, categorical RHIS indicator, flagging discontinuities meriting manual review. It is designed not to generate false confidence through circular corroboration.

For applied machine learning, we highlight one practical recommendation from this case. Before reporting that an independent method corroborates a primary finding, one should list every input feature used by the corroborating method. Each feature should then be checked for a direct or derived relationship to the primary method’s target or ordering variable, which in our case was certification date. This check is simple to state and was still missed in our initial analysis. We suggest this as a standard, low-cost addition to any workflow combining change-point detection with a second, ostensibly independent method. This matters most for small administrative datasets, where a single dominant temporal signal can easily propagate, unnoticed, into every derived feature. This kind of explicit, checkable pre-registration of what a corroborating analysis is allowed to use follows the broader spirit of published guidance on reproducible computational research [26].

### Limitations and future directions

This study has several limitations. The dataset is a single, cross-sectional extraction with no hospital-level covariates (bed size, hospital level, budget, or staffing); the underlying GCH sub-domain scores [14] were unavailable. This restricts our ability to characterize which aspects of hospital practice, if any, the outcome field reflects. The sample is also small (*n* = 86) and specific to one region and one certification program, limiting generalizability of the substantive GCH finding. Because each hospital contributes a single record, the discontinuity reflects compositional change across hospitals at each time point, not change tracked within the same hospitals over time. This limits the strength of any temporal or causal inference drawn from the pattern. The leakage pattern follows from the pipeline structure and general statistical reasoning. Whether the protocol performs well across other RHIS indicators, programs, and settings remains untested. Small samples make these checks important: limited data amplifies estimate uncertainty, and leakage effects are larger and more variable in smaller datasets [27]. The robustness checks are limited to available features; a more direct test would require the GCH sub-domain scores or records of how the outcome field was populated. One administrative record also showed an implausible −1 day certificate duration. We retained it throughout as a flagged likely data-entry error. Excluding it leaves certificate duration for the remaining 85 hospitals confined to a narrow 729–760 day range. Any analysis using this variable’s absolute scale, rather than its within-sample ranking, should account for that concentration.

Future work could extend the protocol to other RHIS indicators and programs, testing how often this leakage pattern recurs. Linking this dataset to hospital-level covariates and GCH sub-domain scores would more directly test whether the discontinuity reflects measurable change in hospital environmental practice. Direct engagement with the Health Region 11 environmental-health office would most directly resolve the specific administrative question this study raises but cannot. A packaged, reusable implementation, taking a timestamped categorical outcome and candidate features as input, would flag leaking features before any corroborating analysis runs and support automated RHIS screening.

## Conclusion

This study demonstrated a previously undocumented data-leakage instance: a clustering analysis appeared to independently corroborate a change-point finding while sharing a date-derived feature with it. In GCH certification records for 86 hospitals in Health Region 11, Thailand this leakage produced a false corroboration for a renewal-outcome discontinuity. We developed a non-circular protocol to detect it, validating the discontinuity against an alternative construct and a clustering analysis restricted to date-unrelated features. Neither check corroborated the original discontinuity, consistent with a recording or definitional change rather than genuine hospital-performance change. A synthetic dataset with a known, genuine, non-leaking discontinuity provided preliminary evidence that the protocol can correctly corroborate a real effect when one is present. To our knowledge, this pipeline type has not been previously covered: change-point detection paired with clustering, extended to routine health administrative data. For regional health offices, the contribution is a low-cost, automatable protocol for manual data-quality review, flagging administrative artifacts before they are reported as real change. A finding should not be validated using information correlated with the finding. The methodological principle illustrated by this study is intended to be general. However, the protocol has been evaluated in only one real case and a preliminary six-scenario synthetic check. Establishing how well it performs across other RHIS indicators, programs, and settings will require external, repeated validation beyond this study.

## Supplementary Material

Supplementary_Tables_revision2_Clean.docx

## Data Availability

The data that support the findings of this study are openly available in Zenodo at https://doi.org/10.5281/zenodo.23754038, reference number 23754038.
