# Supplementary material for: Distinguishing programmatic change from data artifacts in hospital certification records: a change‑point and leakage-audit approach in Thailand
Source: Glob Health Action. 2026 Nov 11;19(1):2758984. doi: 10.1080/16549716.2026.2758984 (PMC13757874; doi:10.1080/16549716.2026.2758984)
Supplement: Supplementary file 1 [file ZGHA_A_2758984_SM0303.docx]

**Supplementary Table S1.** Unit of observation

| **Hospital ID (anonymized, sequential)** | **Province code** | **Certification date** | **Recorded outcome** | **Multiple observations for this hospital?** |
| --- | --- | --- | --- | --- |
| H001 | 86 | 25/07/2023 | Maintained | No (single record) |
| H002 | 80 | 18/08/2023 | Maintained | No (single record) |
| H003 | 86 | 11/09/2023 | Maintained | No (single record) |
| H004 | 86 | 11/09/2023 | Maintained | No (single record) |
| H005 | 81 | 11/09/2023 | Maintained | No (single record) |
| H006 | 83 | 11/09/2023 | Maintained | No (single record) |
| H007 | 82 | 11/09/2023 | Maintained | No (single record) |
| H008 | 80 | 11/09/2023 | Maintained | No (single record) |
| H009 | 85 | 04/01/2024 | Maintained | No (single record) |
| H010 | 82 | 28/02/2024 | Maintained | No (single record) |
| H011 | 81 | 28/02/2024 | Maintained | No (single record) |
| H012 | 83 | 28/02/2024 | Maintained | No (single record) |
| H013 | 80 | 28/02/2024 | Maintained | No (single record) |
| H014 | 82 | 28/02/2024 | Maintained | No (single record) |
| H015 | 82 | 28/02/2024 | Maintained | No (single record) |
| H016 | 81 | 29/02/2024 | Maintained | No (single record) |
| H017 | 80 | 29/02/2024 | Maintained | No (single record) |
| H018 | 84 | 29/02/2024 | Maintained | No (single record) |
| H019 | 80 | 29/02/2024 | Maintained | No (single record) |
| H020 | 84 | 01/03/2024 | Maintained | No (single record) |
| H021 | 84 | 01/03/2024 | Maintained | No (single record) |
| H022 | 81 | 05/03/2024 | Maintained | No (single record) |
| H023 | 80 | 20/03/2024 | Maintained | No (single record) |
| H024 | 80 | 21/03/2024 | Maintained | No (single record) |
| H025 | 82 | 18/04/2024 | Maintained | No (single record) |
| H026 | 82 | 18/04/2024 | Maintained | No (single record) |
| H027 | 86 | 15/07/2024 | Maintained | No (single record) |
| H028 | 86 | 15/07/2024 | Maintained | No (single record) |
| H029 | 84 | 25/07/2024 | Maintained | No (single record) |
| H030 | 80 | 25/07/2024 | Maintained | No (single record) |
| H031 | 82 | 20/09/2024 | Maintained | No (single record) |
| H032 | 84 | 23/11/2024 | Maintained | No (single record) |
| H033 | 81 | 09/01/2025 | Upgraded | No (single record) |
| H034 | 86 | 09/01/2025 | Upgraded | No (single record) |
| H035 | 84 | 09/01/2025 | Upgraded | No (single record) |
| H036 | 84 | 16/01/2025 | Maintained | No (single record) |
| H037 | 84 | 16/01/2025 | Upgraded | No (single record) |
| H038 | 84 | 16/01/2025 | Upgraded | No (single record) |
| H039 | 84 | 16/01/2025 | Maintained | No (single record) |
| H040 | 84 | 16/01/2025 | Maintained | No (single record) |
| H041 | 81 | 16/01/2025 | Maintained | No (single record) |
| H042 | 84 | 16/01/2025 | Upgraded | No (single record) |
| H043 | 84 | 16/01/2025 | Upgraded | No (single record) |
| H044 | 84 | 16/01/2025 | Upgraded | No (single record) |
| H045 | 84 | 16/01/2025 | Upgraded | No (single record) |
| H046 | 84 | 16/01/2025 | Upgraded | No (single record) |
| H047 | 80 | 24/02/2025 | Upgraded | No (single record) |
| H048 | 80 | 24/02/2025 | Upgraded | No (single record) |
| H049 | 80 | 24/02/2025 | Upgraded | No (single record) |
| H050 | 80 | 24/02/2025 | Upgraded | No (single record) |
| H051 | 80 | 24/02/2025 | Upgraded | No (single record) |
| H052 | 80 | 24/02/2025 | Upgraded | No (single record) |
| H053 | 80 | 24/02/2025 | Upgraded | No (single record) |
| H054 | 86 | 27/02/2025 | Upgraded | No (single record) |
| H055 | 81 | 03/03/2025 | Upgraded | No (single record) |
| H056 | 80 | 09/03/2025 | Upgraded | No (single record) |
| H057 | 80 | 19/03/2025 | Upgraded | No (single record) |
| H058 | 80 | 19/03/2025 | Upgraded | No (single record) |
| H059 | 80 | 31/03/2025 | Upgraded | No (single record) |
| H060 | 83 | 26/05/2025 | Upgraded | No (single record) |
| H061 | 84 | 26/05/2025 | Upgraded | No (single record) |
| H062 | 83 | 26/05/2025 | Upgraded | No (single record) |
| H063 | 80 | 02/06/2025 | Upgraded | No (single record) |
| H064 | 85 | 09/06/2025 | Upgraded | No (single record) |
| H065 | 81 | 10/06/2025 | Upgraded | No (single record) |
| H066 | 80 | 11/06/2025 | Upgraded | No (single record) |
| H067 | 80 | 18/06/2025 | Upgraded | No (single record) |
| H068 | 86 | 30/06/2025 | Upgraded | No (single record) |
| H069 | 84 | 02/07/2025 | Upgraded | No (single record) |
| H070 | 85 | 02/07/2025 | Upgraded | No (single record) |
| H071 | 84 | 02/07/2025 | Upgraded | No (single record) |
| H072 | 86 | 02/07/2025 | Upgraded | No (single record) |
| H073 | 84 | 02/07/2025 | Upgraded | No (single record) |
| H074 | 86 | 04/07/2025 | Upgraded | No (single record) |
| H075 | 86 | 04/07/2025 | Upgraded | No (single record) |
| H076 | 84 | 04/07/2025 | Upgraded | No (single record) |
| H077 | 80 | 09/07/2025 | Upgraded | No (single record) |
| H078 | 82 | 09/07/2025 | Upgraded | No (single record) |
| H079 | 85 | 14/07/2025 | Maintained | No (single record) |
| H080 | 84 | 15/07/2025 | Upgraded | No (single record) |
| H081 | 82 | 15/07/2025 | Upgraded | No (single record) |
| H082 | 81 | 23/07/2025 | Upgraded | No (single record) |
| H083 | 80 | 23/07/2025 | Upgraded | No (single record) |
| H084 | 84 | 31/07/2025 | Upgraded | No (single record) |
| H085 | 85 | 31/07/2025 | Upgraded | No (single record) |
| H086 | 84 | 31/07/2025 | Upgraded | No (single record) |
| H001 | 86 | 25/07/2023 | Maintained | No (single record) |

**Supplementary Table S2.** Synthetic scenarios summary

| **Scenario** | **n_replicates** | **pct_breakpoint_found** | **pct_corroborated_of_found** |
| --- | --- | --- | --- |
| none | 50 | 0 | n/a |
| genuine | 50 | 100 | 84 |
| artifact | 50 | 100 | 0 |
| gradual | 50 | 80 | 85 |
| multi_cp | 50 | 100 | 92 |
| small_n | 50 | 52 | 50 |
